# Immune checkpoint molecules soluble program death ligand 1 and galectin‐9 are increased in pregnancy

**DOI:** 10.1111/aji.12795

**Published:** 2017-12-04

**Authors:** Elizabeth Ann L. Enninga, Susan M. Harrington, Douglas J. Creedon, Rodrigo Ruano, Svetomir N. Markovic, Haidong Dong, Roxana S. Dronca

**Affiliations:** ^1^ Department of Obstetrics and Gynecology Mayo Clinic Rochester MN USA; ^2^ Department of Urology Mayo Clinic Rochester MN USA; ^3^ Department of Obstetrics and Gynecology Baylor College of Medicine at the Children's Hospital of San Antonio San Antonio TX USA; ^4^ Department of Medical Oncology Mayo Clinic Rochester MN USA

**Keywords:** galectin‐9, regulation, sPD‐L1, tolerance

## Abstract

**Problem:**

Pregnancy requires balance between tolerance to the haploidentical fetus and the mother's ability to mount immune responses. There are parallels to this phenomenon that occur in metastatic cancer. We assessed soluble program death ligand‐1 soluble PD‐L1 (sPD‐L1) and galectin‐9 in the blood of pregnant women during gestation as these molecules are highly involved in immune suppression during cancer.

**Method of study:**

Maternal blood was collected from 30 primigravida women at monthly intervals during pregnancy, delivery and 6‐week post‐partum. Blood was analyzed for sPD‐L1 and galectin‐9 concentrations by ELISA. Term placentas were collected in formalin and IHC was completed for PD‐L1 and galectin‐9 expression.

**Results:**

Maternal blood levels of sPD‐L1 (0.438 ng/mL) and galectin‐9 (1976 pg/mL) were elevated early in normal pregnancies compared to non‐pregnant controls (0.242 ng/mL and 773 pg/mL, respectively). sPD‐L1 increased throughout gestation, whereas galectin‐9 remained elevated until parturition; both proteins returned to control levels post‐partum. Women carrying male fetuses had significantly higher galectin‐9 levels, but not sPD‐L1, than those carrying females (2263 pg/mL vs 1874 pg/mL; *P *= .0005). Trophoblast cells of the term placenta coexpress galectin‐9 and PD‐L1.

**Conclusion:**

Immune‐regulatory molecules galectin‐9 and sPD‐L1 increased during pregnancy and may play a role in immune tolerance that is critical for the fetus.

## 1 INTRODUCTION

Defects in regulation of T‐cell homeostasis between the mother and the developing fetus have been recently postulated to be associated with pregnancy‐related complications such as implantation failure, recurrent spontaneous abortions, and pre‐eclampsia.^1^, ^2^ Human reproduction is a delicate balance between immune tolerance and activation that, if disrupted, can result in negative pregnancy outcomes such as preterm birth and miscarriage.^3^ Additionally, gestational complications have been linked to the sex of the fetus.^4^ Thus, a clearer understanding of how this process is regulated could provide a wealth of knowledge useful for treating pregnancy complications.

Recent studies have identified the program death (PD)‐1/PD‐L1 pathway as critical in regulating T‐cell homeostasis and peripheral tolerance in malignancy, and similar pathways are thought to be required for tolerance of the developing fetus in pregnancy. PD‐L1 promotes T regulatory cell development and function and directly inhibits effector T‐cell differentiation, cytokine production, and target cell lysis.^5^ This pathway has been demonstrated to play an important role in tumour‐induced immunosuppression in melanoma and other advanced malignancies and now is being targeted as a therapeutic.^6^, ^7^, ^8^, ^9^ Anti‐ program death‐1(PD‐1) antibodies restore antitumour immunity by impeding interactions of PD‐1 receptor expressed by tumour‐reactive T‐cells with PD‐1 ligands (such as PD‐L1/B7‐H1) expressed by tumour cells.^8^ Membrane bound B7‐H1 (PD‐L1) was discovered in 1998 by cloning the molecule from the human placenta.^10^ A decade later, the soluble version of PD‐L1 (sPD‐L1) was identified in sera of patients with cancer is biologically active and capable of triggering apoptotic signals in target T‐cells due to retention of PD‐1‐binding domain.^11^ The release of biologically active sPD‐L1 into the circulation would predictably lead to T‐cell PD‐1 receptor engagement with its ligand PD‐L1 at the tumour site but also systemically. Therefore, in patients with cancer, sPD‐L1 may represent an unanticipated contributing factor to the overall tumour‐induced immune suppression and acts by inhibiting circulating immune T‐cells even beyond the tumour microenvironment. The PD‐1/PD‐L1 interaction is also important for maintenance of normal pregnancy.^12^ Treatment with anti‐PD‐L1‐blocking antibody resulted in spontaneous resorption in murine allogeneic pregnancy model.^13^ However, pregnant mice with genetic depletion of PD‐1 or PD‐L1 had normal litters, demonstrating that maternal tolerance is not solely dependent on this pathway.^14^

Galectin‐9 is another molecule with a role in inflammation, development, and malignancy that may have a similar role in pregnancy.^15^, ^16^ In metastatic melanoma, high soluble galectin‐9 is correlated with T helper 2 polarization and monocyte differentiation toward an M2 tumour‐promoting phenotype.^17^ Much of the research on galectin‐9 has focused on its interaction with T‐cell immunoglobulin mucin domain‐3 (Tim‐3) receptor, which effectively inhibits T helper 1 immune responses.^18^ In pregnancy, galectin‐9 is secreted by trophoblast cells and can cause Tim3 +  peripheral natural killer cells to take on a decidual NK (dNK) phenotype defined by high levels of interleukin (IL)‐4, low levels of tumour necrosis factor (TNF)‐α, and decreased cytotoxic capabilities.^19^, ^20^ We hypothesize that galectin‐9 and sPD‐L1 will be elevated in the blood of women having uncomplicated pregnancies to protect the fetus from the maternal immune system.

## 2 METHODS

This study was approved by the Mayo Clinic Institutional Review Board. Ten milliliters of peripheral blood was collected from 30 consenting primigravida women with healthy, uncomplicated pregnancies. Collections were monthly starting at 8 weeks, and then 2‐hour post‐delivery and 6‐weeks post‐partum (n = 10 average time points per person). A section of the placenta was also collected at the time of delivery, fixed in formalin, and embedded in paraffin for immunohistochemistry. Blood was also collected from 15 non‐pregnant, reproductive age females undergoing blood donation at Mayo Clinic. Whole blood was processed to plasma and buffy coats were isolated using Ficoll‐Paque gradients (GE Healthcare Bio‐Sciences, Pittsburgh, PA, USA). Plasma was stored at −80°C until use.

To measure plasma levels of galectin‐9, DuoSet ELISA antibodies were utilized as per the manufacture's instruction (R&D Systems, Minneapolis, MN, USA). For sPD‐L1, a sandwich ELISA was developed using paired mouse monoclonal antibodies (H1A and B11) raised against the extracellular domain of human PD‐L1. The specificity of each antibody was validated by immunohistochemistry and flow cytometry. Both antibodies bind to the extracellular domain of PD‐L1 and to different sites on the PD‐L1 molecule. The assay is specific for PD‐L1 and does not exhibit cross‐reactivity to other B7‐H homologues (B7‐H2, B7‐H3, B7‐H4 or PD‐1; all from R&D Systems), or third‐party recombinant protein P‐selectin (R&D Systems). Binding of H1A to PD‐L1 in the ELISA can be blocked by pre‐incubation of standards with antibody (Figure S1 A,B). H1A was used as the plate‐fixed capture antibody and biotinylated B11 was used as the detection antibody. Biotinylation was performed using a solid‐phase kit (ThermoFisher, Waltham, MA, USA). Individual ELISA steps are followed by 3 washes, using a PBS + 0.05% Tween‐20 buffer. High‐binding polystyrene plates were coated for 2 hours at 21°C with 0.1 ug per well of H1A. Free binding sites were blocked with 200 uL per well of Superblock (ThermoFisher) for 1 hour at 21°C. After washing, 50 uL of samples and standards were added to 50 uL of assay buffer (PBS + 1% bovine serum albumin) and incubated at 4°C overnight. Biotinylated B11 (100 uL per well at 1 ug/mL diluted in PBS + 0.1% bovine serum albumin) was added and incubated for 1 hour at 21°C. After washing, 100 uL of horseradish peroxidase‐conjugated streptavidin (BD Biosciences, San Jose, CA, USA) diluted in PBS + 0.1% bovine serum albumin was added and incubated for 1 hour at 21°C. Plates were developed with tetramethylbenzidine (ThermoFisher), stopped using 0.5 Sulfuric Acid and read at 450 nm with a Benchmark Plus plate reader and associated software (BioRad, Hercules, CA, USA). For calibration, each plate was loaded with parallel dilutions of recombinant PD‐L1 fusion protein (R&D Systems), ranging in concentration from 0.009 to 20 ng/mL.

Ten term placentas were fixed in formalin and embedded in paraffin. Antibodies against galectin‐9 (AbCam, Cambridge, MA, USA) or PD‐L1 (Clone E1L3N; Cell Signaling, Danvers, MA, USA) were utilized for staining, along with isotype controls. The galectin‐9 antibody was diluted 1:500 for immunohistochemistry while the PD‐L1 antibody was diluted 1:400 for staining.

All samples were run in duplicate and averaged. Data are presented in means and standard error of the mean (SEM). Statistical significance was determined using a 2‐tailed student's *t* test. Longitudinal data were measured by ANOVA, and compared to post‐partum concentrations. *P*‐values < .05 were considered significant.

## 3 RESULTS

### 3.1 Patient characteristics

All women included in our pregnant cohort were primigravida and ranged in age from 19 to 34 years with an average age of 28.4. They were non‐smokers and had no known infections, autoimmune disease or prior cancer diagnoses. Of this cohort, 15 women carried and delivered male offspring and 15 had females. This occurrence further permitted a direct comparison of infant sex on the levels of the factors assayed in this study. The control cohort was comprised of reproductive age, non‐pregnant women who were blood donors at Mayo Clinic. The non‐pregnant women in the control population ranged in age from 22 to 44 years, with an average age of 36.8 years. All patients in the control group had negative pregnancy tests at the time of blood collection, but pregnancy or other history was not collected for these subjects.

### 3.2 Soluble PD‐L1 and galectin‐9 levels increase during normal gestation

**Figure 1:**
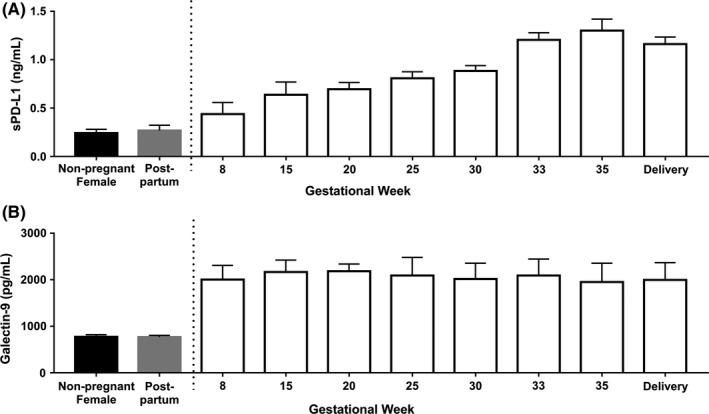
sPD‐L1 and galectin‐9 are increased in the maternal blood during normal pregnancies. A, sPD‐L1 concentrations increase steadily throughout gestation, beginning to drop right before delivery and are elevated during normal pregnancies compared to post‐partum and non‐pregnant females. B, Galectin‐9 concentrations are significantly higher throughout gestation in women with normal pregnancies compared to post‐partum and non‐pregnant female controls. sPD‐L1, soluble PD‐L1

Elevations in both sPD‐L1 (Figure 1A) and galectin‐9 (Figure 1B) were evident at the first collection time‐point. At 8 weeks, sPD‐L1 levels were not statistically different from non‐pregnant controls (0.438 ng/mL ±0.12 vs 0.157 ng/mL ±0.33; *P *= .45). However, by 15 weeks, sPD‐L1 levels were significantly higher than non‐pregnant controls (0.638 ng/mL ±0.13; *P* = .005). On the other hand, galectin‐9 levels were nearly 3 times non‐pregnant levels at 8 weeks of gestation (2003 pg/mL ±304 vs 773 pg/mL ±46; *P* < .0001). Both molecules had returned to control levels by 6‐week post‐partum (sPD‐L1: 0.269 ng/mL ±0.05, *P* = .99; galectin‐9: 769 pg/mL ±35, *P* = .99). Table 1 demonstrates the mean differences measured between each time point and compared to the post‐partum levels of galectin‐9 and sPD‐L1.

**Table 1:** ANOVA results of longitudinal differences between sPD‐L1 and galectin‐9 concentrations in gestation compared to post‐partum

Gestational week	sPD‐L1			Galectin‐9		
	Mean difference	SEM	*P*‐value	Mean difference	SEM	*P*‐value
8 vs Post‐partum	0.067	0.08	.451	1100	364	.0002
15 vs Post‐partum	0.266	0.08	.005	1263	364	.0001
20 vs Post‐partum	0.324	0.08	.001	1281	364	.0001
25 vs Post‐partum	0.438	0.08	.0001	1186	364	.0001
30 vs Post‐partum	0.513	0.08	.0001	1112	364	.0001
33 vs Post‐partum	0.809	0.09	.0001	1189	364	.0001
35 vs Post‐partum	0.93	0.08	.0001	1049	364	.0003
40 vs Post‐partum	0.791	0.08	.0001	1093	364	.0002

sPD‐L1, soluble PD‐L1.

### 3.3 Placentas express high levels of sPD‐L1 and galectin‐9

**Figure 2:**
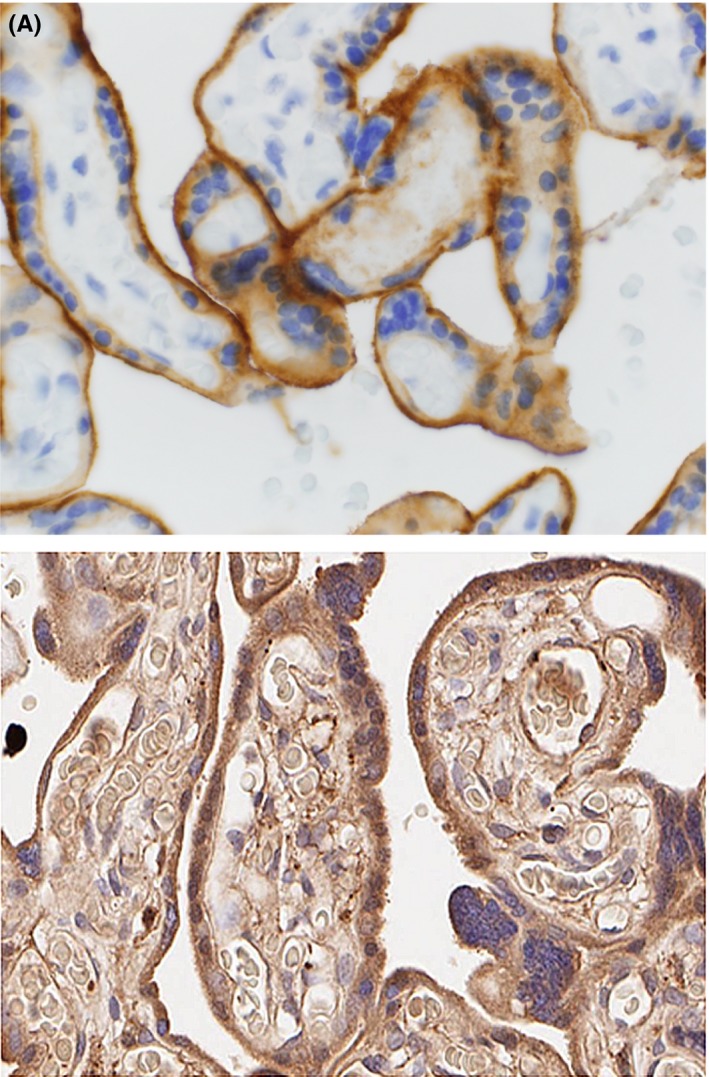
Term placentas highly express negative regulatory proteins. A, PD‐L1 expression on trophoblasts. B, Galectin‐9 expression on trophoblast cells

Immunohistochemical studies confirmed that PD‐L1 is highly expressed on trophoblast cells of the term placenta (Figure 2A). Term placentas also express high levels of galectin‐9 around the trophoblast cells (Figure 2B). Unlike PD‐L1 expression, galectin‐9 can be detected both intracellularly and membrane bound. Our results suggest that sPD‐L1 and galectin‐9 are highly expressed on cells at the fetal‐maternal interface.

### 3.4 Differences in sPD‐L1 and galectin‐9 based on the sex of the fetus

**Figure 3:**
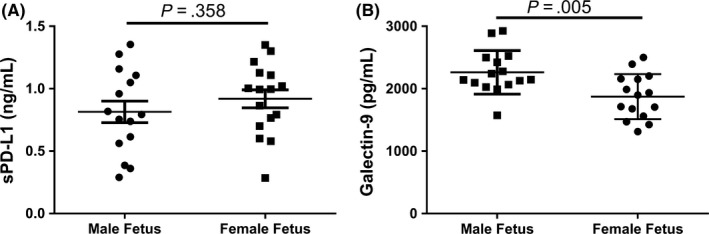
Differences in the maternal blood concentration of sPD‐L1 and galectin‐9 based on the sex of the fetus. A, No differences in sPD‐L1 levels in between pregnancies with a male vs female fetus. B, Galectin‐9 is increased further in the blood of women carrying a male fetus vs a female fetus. sPD‐L1, soluble PD‐L1

There were no significant differences in sPD‐L1 levels based on whether the fetus was a female or male (0.815 ng/mL ±0.086 vs 0.944 ng/mL ±0.082, respectively; *P* = .358) (Figure 3A). Interestingly, women carrying male fetuses had elevated blood levels of galectin‐9 compared to women carrying female fetuses (2263 pg/mL ±89.6 vs 1874 pg/mL ±92.9, respectively; *P* = .005) (Figure 3B).

## 4 DISCUSSION

Our data support the hypothesis that proteins with immune regulating capabilities are important for a healthy pregnancy and that the placenta may use sPD‐L1 and galectin‐9 to neutralize systemic maternal T‐cells against the haploidentical fetus. This role would be analogous to that of PD‐L1 in metastatic tumours. PD‐L1 (aka B7‐H1, CD274) was originally cloned from a cDNA library of the human placenta and was predicted to function as an organ‐specific negative regulator of cellular immune responses.^10^ Constitutive PD‐L1 protein expression is demonstrated at the syncytiotrophoblast and extravillous cytotrophoblasts, both of which are juxtaposed to the maternal blood and tissue,^21^ suggesting that PD‐L1 may function as a barrier to prevent the fetus from allogeneic immune responses.^22^, ^23^ Interestingly, PD‐L1 protein expression increased from first‐trimester placenta through second‐ and third‐trimester tissues, and has been found in placental exosomes.^21^, ^24^ In line with this observation, our study shows that maternal sPD‐L1 levels were low at 8 weeks of gestation, but gradually increased until right before delivery and then began dropping to normal levels 2‐hour post‐partum. Future research should be conducted to determine if sPD‐L1 is packaged within microvesicles, and whether packaging changes the function or effect of these immunomodulatory proteins. Studies have shown that IFN‐γ and epidermal growth factor enhance PD‐L1 expression in cultured cytotrophoblasts;^21^ therefore, the increase in sPD‐L1 in the maternal blood likely indicates an ongoing counter balance of pro‐inflammatory and immune tolerance during gestation along with the rapid growth of the allogenic fetus.

Studies of galectin biology have demonstrated numerous roles for this family of proteins in inflammation, infection, cancer, development, and signaling.^15^, ^16^ Galectin‐9 was found to bind Tim3 on T helper 1 cells and initiate apoptosis to deescalate immune responses.^18^ Once this interaction was defined, cancer biologists demonstrated that mice who developed a resistance to anti‐PD‐1 therapy responded to anti‐Tim3‐blocking antibody, leading to improved survival.^25^ However, other studies have found that Tim3 is not necessary for galectin‐9 to promote immune tolerance and cellular apoptosis.^17^, ^26^ While the role of Tim3/galectin‐9 in cancer remains in debate, the outcome of this interaction could have important implications in human pregnancy. During normal gestation, Meggyes et al^27^ revealed galectin‐9 increased with each trimester; however, the expression of Tim3 on maternal cytotoxic cells and NK cells did not change. The current study complements this previous study, and adds additional time points to demonstrate that the maternal blood levels of galectin‐9 remain elevated throughout gestation.

We found that both galectin‐9 and sPD‐L1 levels increase with length of gestation. As the placenta highly expresses both immunomodulatory proteins, this increase may be due to the growth of the placenta through pregnancy; however, the placenta is not the only source of these proteins. Throughout the maturation process, dendritic cells release increasing amounts of sPD‐L1 which can initiate apoptosis of CD4 +  and CD8 +  T‐cells as a mechanism of immune homeostasis.^28^ CD4 +  cells have been shown to secrete galectin‐9 upon T‐cell receptor activation, but this mechanism remains unclear.^29^ Using human myeloid cell lines, researchers showed that these cells secrete high concentrations of galectin‐9 with and without stimulation, and that secretion was dependent on Tim3.^30^ Therefore, the contribution of soluble galectin‐9 and sPD‐L1 by maternal immune cells during gestation warrants further study.

If the role of sPD‐L1 and galectin‐9 during pregnancy is to modulate maternal immune tolerance to support fetal growth and development, it is important to consider their role in spontaneous miscarriage or recurrent pregnancy loss. Studies have demonstrated that mRNA expression of PD‐L1, but not PD‐1, is significantly decreased in decidual tissues from cases of recurrent miscarriage compared to controls.^31^ Similarly, Wu et al^32^ found that women with recurrent spontaneous abortions (defined as 3 or more pregnancy losses) had lower levels of galectin‐9 in peripheral blood compared to the normal pregnancy group. In addition, placentas from fetal resorption models had lower mRNA quantities of galectin‐9 compared to normal placenta.^33^ Thus, further studies are necessary to determine whether this could serve as a biomarker to assess the health of the pregnancy or whether exogenous galectin‐9 might be used to prevent pregnancy loss.

The sex of the fetus is another important variable which must be considered when studying fetal‐maternal interactions. Many pregnancy outcomes have been associated with the sex of fetus; for example, male fetuses have an increased incidence of preterm birth and gestational diabetes, whereas mothers carrying female fetuses more often present with hypertensive disorders.^4^ In the current study, blood levels of galectin‐9, but not sPD‐L1, were significantly higher in pregnancies with male fetuses compared to females. Studies of the placenta indicated that inflammation was more common in obese mothers with female fetuses compared to male fetuses.^34^ Systemically, male fetuses induce a more inflammatory state in the maternal blood during pregnancy than females.^35^ On the contrary, it has recently been demonstrated that female fetuses induce a more robust inflammatory response to bacterial stimulus during pregnancy compared to male fetuses.^36^ However, male antigens are not the only epitopes recognized by the maternal immune system. HLA‐G G*0106 variant contributed by the father has been shown to increase the risk for preeclampsia in multigravida pregnancies irrespective of sex.^37^ Other factors, such as differences in endocrine and vascular physiology, likely have important roles in regulating fetal‐maternal interactions between male and female fetuses.^4^, ^38^ Taken together with our current results, these data suggest that maternal immune responses are variable based on the sex of the fetus, requiring researchers to consider fetal sex in experimental design.

Our results support the relevance of the PD‐1/PD‐L1 and galectin‐9 pathway in fetal‐maternal tolerance and pregnancy‐related complications. Therefore, manipulation of these pathways may represent a novel approach to maintaining and enhancing maternal tolerance in pregnancy. Currently, although anti‐PD‐1 and anti‐PD‐L1 antibodies have become routine in oncology care, there are no tools to block the action of galectin‐9 for humans. Further studies are still needed to better understand the potential role of these molecules in pregnancy, especially in pregnancy loss. Continued collaborations between clinicians and scientists, especially those made up of multidisciplinary teams, will provide us with creative new ways to understand complicated biology and improve outcomes in obstetrics and oncology.

## CONFLICT OF INTEREST

H. Dong and S. Harrington are the inventors of U.S. patent application WO2015050663A1, and H. Dong and S. Markovic hold US patent 9302005 B2. The authors have no other relevant affiliations or financial involvement with any organization or entity having financial interest in or financial conflict with the subject matter or materials discussed in the manuscript apart from those disclosed.
