# Supplementary figures and images for: Immune checkpoint molecules soluble program death ligand 1 and galectin‐9 are increased in pregnancy
Source: Am J Reprod Immunol. 2017 Dec 4;79(2):e12795. doi: 10.1111/aji.12795 (PMC5814874; doi:10.1111/aji.12795)

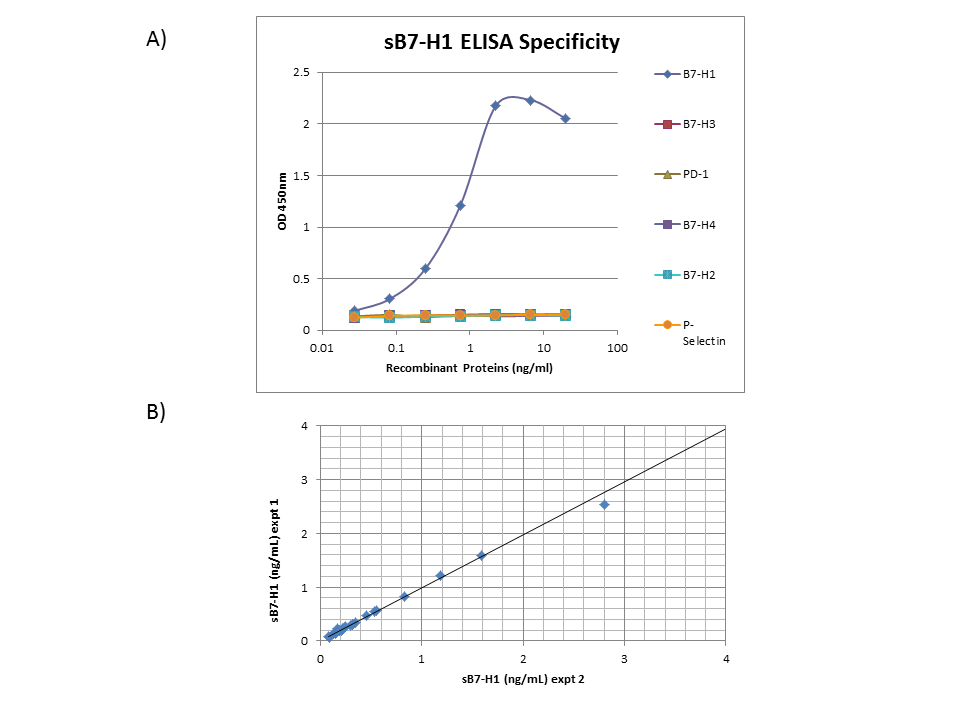

Supplement: Supplementary file 1 [file AJI-79-na-s001.tif]
